# Epidemiological Characteristics, Species Distribution, and Antifungal Resistance Profiles of Candidemia Cases

**DOI:** 10.3390/jof12100715

**Published:** 2026-09-23

**Authors:** Demet Timur, Emel Gürcüoğlu, Emre Özkarataş, Tuğba Bozdemir

**Affiliations:** 1 Medical Microbiology Laboratory, Bursa City Hospital, Doğanköy Mahallesi, Şifa Caddesi No: 1, 16250 Nilüfer, Bursa, Türkiye; 2 Department of Infectious Diseases, Bursa City Hospital, Doğanköy Mahallesi, Şifa Caddesi No: 1, 16250 Nilüfer, Bursa, Türkiye

**Keywords:** candidemia, *Candida* species, antifungal susceptibility, *Candida parapsilosis*, persistent candidemia, recurrence, mortality

## Abstract

Local species distribution and antifungal susceptibility are important for empirical candidemia management. We retrospectively evaluated patients with *Candida*-positive blood cultures from August 2019 to May 2024. Episodes were defined using a 30-day window; species distribution and susceptibility were analysed at the episode–species level and outcomes at the patient level. Among 566 patients, 605 episodes and 637 episode–species isolates were identified. Non-albicans *Candida* accounted for 59.7%; *C. albicans* (40.3%) and *C. parapsilosis* (34.2%) predominated. Of 217 *C. parapsilosis* isolates tested, 66 (30.4%) were fluconazole-susceptible, 58 (26.7%) were susceptible dose-dependent, and 93 (42.9%) were resistant. Recurrent candidemia occurred in 37 patients and documented microbiological persistence in 128 (22.6%); persistence was more frequent when the index culture included *C. parapsilosis* (27.6% vs. 20.1%; OR 1.52, 95% CI 1.01–2.28). Among 32 patients with a shared species in the initial and first recurrent episodes, newly detected resistance occurred in three, all involving fluconazole-resistant *C. parapsilosis*. Thirty-day all-cause mortality was 57.4%; older age, ICU care, mechanical ventilation, and solid malignancy were independently associated with higher mortality. These findings suggest that empirical reliance on fluconazole may be inappropriate in settings with similar resistance patterns and support species-level identification, repeat blood cultures, and episode-specific susceptibility testing.

## 1. Introduction

Candidemia remains an important cause of morbidity and mortality, particularly in patients with severe underlying diseases or those requiring intensive care [1,2]. Broad-spectrum antibiotic use, central venous catheterisation, parenteral nutrition, mechanical ventilation, previous gastrointestinal surgery, prolonged hospitalisation, immunosuppression, malignancy, and intensive care unit stay are among the clinical conditions associated with candidemia [3].

The epidemiology of candidemia varies according to geographic region, hospital setting, and patient population [3,4,5]. Although *Candida albicans* remains one of the most frequently isolated species, non-albicans *Candida* species have become increasingly important in many centres [1,3,4,5]. This shift has clinical implications because *Candida* species differ in their antifungal susceptibility profiles, particularly to azole agents [4,5].

Among non-albicans *Candida* species, *C. parapsilosis* is particularly relevant as a cause of invasive candidiasis, and its proportion among candidemia cases has increased over time [6]. Its association with invasive medical devices, particularly central venous catheters, and the reduced antifungal susceptibility of biofilm-associated cells contribute to its clinical importance [6]. In parallel, increasing azole resistance and the emergence of different mechanisms of resistance to azoles and echinocandins have raised growing clinical concern [6,7].

Therefore, species-level identification, antifungal susceptibility testing, and periodic evaluation of local data are important in candidemia management. Local surveillance may help guide empirical treatment decisions and support antifungal stewardship and infection control practices [4,5]. This study evaluated the epidemiological characteristics, species distribution, antifungal susceptibility profiles, recurrent and mixed-species episodes, documented persistent candidemia, and mortality outcomes of candidemia cases in our hospital.

## 2. Materials and Methods

In this retrospective study, patients who were hospitalised at Bursa City Hospital between August 2019 and May 2024 and whose blood cultures yielded *Candida* species were included.

During the study period, blood culture bottles flagged as positive by the automated blood culture system were processed in accordance with the routine procedures of the clinical microbiology laboratory. Direct Gram staining was performed from positive bottles, and samples were inoculated onto 5% sheep blood agar and eosin methylene blue (EMB) agar plates. Culture plates were incubated at 37 °C for 24 h; when necessary, the incubation period was extended to 48 h.

When yeast growth was detected in blood cultures, a germ tube test was performed as the initial step. Isolates were also inoculated onto chromogenic agar and corn meal agar for phenotypic and micromorphological evaluation and incubated at 30 °C for 24–48 h. All culture media were obtained from RTA Laboratories (Gebze, Türkiye).

Yeast colonies grown on sheep blood agar were identified to the species level using the VITEK^®^ 2 YST card (bioMérieux, Marcy-l’Étoile, France). The two isolates identified as *C. auris* by VITEK 2 were further confirmed by MALDI-TOF MS (Bruker Daltonics, Bremen, Germany). Antifungal susceptibility testing was performed with the VITEK^®^ 2 AST-YS08 card on the automated VITEK^®^ 2 system (bioMérieux, Marcy-l’Étoile, France), in accordance with the manufacturer’s recommendations. Available antifungal susceptibility results for fluconazole, voriconazole, micafungin, and amphotericin B were retrospectively evaluated and interpreted according to the Clinical and Laboratory Standards Institute (CLSI) criteria. Interpretive categories were assigned from the MIC values generated by the VITEK 2 AST-YS08 card using CLSI clinical breakpoints or, where no clinical breakpoint was available, epidemiological cutoff values, rather than the interpretive categories reported by the instrument. Quality control was performed using the reference strains *Candida parapsilosis* ATCC 22019 and *Candida krusei* ATCC 6258 (ATCC, Manassas, VA, USA; currently named *Pichia kudriavzevii*). For species with revised taxonomic nomenclature, the commonly used *Candida* names were retained alongside the current accepted names to maintain clinical familiarity and taxonomic clarity [8].

Each positive blood culture barcode record was evaluated as a separate culture record. When more than one *Candida* species was recovered from the same blood culture bottle, the same blood culture set, or repeat blood cultures from the same patient, each species was recorded separately. A candidemia episode was defined as one or more blood cultures yielding *Candida* species in the same patient within a 30-day period. Positive blood cultures obtained more than 30 days after the first positive culture were considered a separate episode. For patient-level analyses, each patient was included once using the initial 30-day candidemia episode. Species involvement in patient-level analyses was defined by identification of the species at any time during the initial episode, except where the index culture was explicitly used to define exposure in the 30-day mortality and persistence analyses. The index culture was defined as the first positive blood culture of the initial episode. Species distribution was evaluated at the episode–species level, including the first isolate of each distinct *Candida* species in each initial or recurrent episode. For antifungal susceptibility analyses, only the susceptibility result of the first isolate of each distinct *Candida* species within each initial or recurrent episode was used.

Documented persistent candidemia was assessed separately for each *Candida* species within each candidemia episode. It was defined as re-isolation of the same *Candida* species from a blood culture obtained on a different calendar date, at least five calendar days after the first isolation of that species within the same 30-day episode. Both initial and recurrent candidemia episodes were evaluated. A patient meeting this criterion for any *Candida* species in any episode was classified as having documented persistent candidemia and was counted only once at the patient level. When more than one *Candida* species met the persistence criterion in the same patient, all persistent species were recorded separately. This culture-date-based approach is consistent with a recent cohort in which persistent candidemia was defined as Candida-positive blood cultures persisting for ≥5 days [9]. Because exact dates of active antifungal treatment initiation and systematic negative follow-up blood cultures were unavailable, this definition was considered a descriptive microbiological outcome rather than persistence defined in relation to active antifungal therapy. Patients without a subsequent positive culture were not assumed to have achieved bloodstream clearance.

Data were obtained retrospectively from the hospital information system. ICU status was defined according to whether the patient was receiving care in an intensive care unit at the time of collection of the index blood culture of the initial candidemia episode. The recorded variables included age, sex, clinical department or ward, isolated *Candida* species, antifungal susceptibility results, underlying diseases and related clinical conditions, invasive procedures and supportive treatments, exposure to broad-spectrum antibiotics before candidemia, antifungal treatment status, and mortality outcomes. Underlying diseases and clinical conditions were categorised as cerebrovascular disease, diabetes mellitus, COVID-19, solid malignancy, haematological malignancy, respiratory disease, cardiovascular disease, trauma, burn injury, prematurity, chronic kidney disease, chronic liver disease, and other diseases. Previous gastrointestinal surgery, percutaneous endoscopic gastrostomy use, central venous catheterisation, urinary catheterisation, total parenteral nutrition, and mechanical ventilation were recorded as clinical exposures and invasive or supportive interventions. All-cause mortality was assessed at 7, 14, 30, and 60 days after the first positive blood culture.

### 2.1. Statistical Analysis

Categorical variables were reported as numbers and percentages, while continuous variables were summarised using medians and interquartile ranges. Categorical comparisons were assessed using the chi-square test or Fisher’s exact test, as appropriate. Annual species distributions were summarised descriptively because 2019 and 2024 represented partial calendar years and episode–species observations were not independent at the patient level. For species-specific descriptive and exploratory analyses, *C. parapsilosis* involvement was defined by its identification at any time during the initial 30-day candidemia episode. In contrast, for the 30-day mortality analysis, *C. parapsilosis* status was defined according to its presence in the index culture, because this analysis required a baseline exposure defined at candidemia onset. There were no missing data for the covariates included in the multivariable models; therefore, no imputation or exclusion due to missing covariate data was required. Model discrimination was assessed using the area under the receiver operating characteristic curve (C-statistic), and calibration was evaluated using the Hosmer–Lemeshow goodness-of-fit test with 10 risk groups.

Among patients with *C. parapsilosis* involvement in the initial candidemia episode and an available fluconazole susceptibility result, isolates classified as susceptible dose-dependent (SDD) or resistant were grouped as fluconazole-non-susceptible, whereas isolates classified as susceptible were considered fluconazole-susceptible. Clinical characteristics were compared between the fluconazole-susceptible and fluconazole-non-susceptible groups. Factors potentially associated with fluconazole non-susceptibility were evaluated using multivariable logistic regression including age, central venous catheterisation, total parenteral nutrition, and previous gastrointestinal surgery. Thirty- and 60-day all-cause mortality were also compared between the two fluconazole susceptibility groups.

Among patients with recurrent candidemia, changes in antifungal susceptibility were evaluated by comparing species shared between the initial and first recurrent episodes. Patients were included in this paired shared-species analysis when at least one *Candida* species was recovered in both episodes and comparable susceptibility results were available for that species. Resistance was considered newly detected when a shared species was classified as resistant or non-WT in the recurrent episode but not in the initial episode. Susceptible dose-dependent or intermediate results alone were not classified as resistance in this analysis.

Factors associated with 30-day all-cause mortality in the overall cohort were examined using univariable and multivariable logistic regression analyses, and the results were expressed as odds ratios with 95% confidence intervals. The multivariable model included age, ICU care at the time of the index blood culture, mechanical ventilation, solid malignancy, antifungal treatment, and the presence of *C. parapsilosis* in the index culture of the initial episode. A *p* value below 0.05 was considered statistically significant.

As an exploratory species-specific analysis, clinical characteristics potentially associated with *C. parapsilosis* involvement in the initial candidemia episode were evaluated using multivariable logistic regression. *C. parapsilosis* involvement was defined as identification of the species at any time during the initial 30-day episode, and each patient was included only once. The multivariable logistic regression model included age, sex, ICU care at the time of the index blood culture, total parenteral nutrition, percutaneous endoscopic gastrostomy, burn injury, solid malignancy, cerebrovascular disease, and central venous catheter use.

Given the clinical and epidemiological relevance of *C. parapsilosis* in candidemia, an exploratory patient-level analysis was performed to evaluate whether documented persistent candidemia differed according to *C. parapsilosis* involvement. The frequency of documented persistent candidemia, assessed across all initial and recurrent episodes, was compared between patients whose index culture of the initial episode did and did not include *C. parapsilosis* using Fisher’s exact test. The association was reported as an odds ratio with a 95% confidence interval. Statistical analyses were performed using IBM SPSS Statistics, version 27.0 (IBM Corp., Armonk, NY, USA).

### 2.2. Generative AI-Assisted Manuscript Preparation

ChatGPT (GPT-5.6; OpenAI) was used during manuscript preparation for English-language editing and limited assistance with the presentation of selected statistical analyses and results. All analyses, interpretations, numerical results, references, and final manuscript content were independently reviewed and verified by the authors.

## 3. Results

During the study period, *Candida* growth was detected in 1007 unique blood culture barcode records obtained from 566 patients. Two different *Candida* species were recovered from the same blood culture barcode in 34 records. When each *Candida* species was counted separately, 1041 barcode–organism entries were identified.

Using the 30-day definition, 605 candidemia episodes were identified, comprising 566 initial and 39 recurrent episodes. Recurrent candidemia occurred in 37 patients: 35 patients had two episodes and 2 patients had three episodes.

Documented persistent candidemia was identified in 128 of 566 patients (22.6%). It was significantly more frequent among patients with index-defined *C. parapsilosis* presence than among those without index-defined *C. parapsilosis* presence (53/192, 27.6% vs. 75/374, 20.1%; OR 1.52, 95% CI 1.01–2.28; Fisher’s exact *p* = 0.044).

At the patient–species level, documented persistence involved *C. parapsilosis* in 54 patients, *C. albicans* in 53, *C. tropicalis* in 14, *C. glabrata* in nine, *C. krusei* in two, and *C. lusitaniae* in one. Five patients met the persistence criterion for two different *Candida* species; therefore, these patient–species counts totalled 133, although 128 unique patients had documented persistent candidemia.

Different *Candida* species were detected within the same 30-day episode in 32 patients. In 18 patients, two species were recovered from the same blood culture barcode on at least one occasion. Some of these patients had more than one such mixed-species blood culture during the same episode, resulting in a total of 34 blood culture barcodes containing two *Candida* species. In the remaining 14 patients, different species were recovered from separate blood culture barcodes within the same episode.

Among the 37 patients with recurrent candidemia, the median interval between the initial and second episodes was 46 days (IQR, 35–79; range, 31–539 days). Thirty patients (81.1%) experienced their second episode within 31–90 days, three (8.1%) within 91–150 days, and four (10.8%) after more than 150 days.

Among the 37 patients with recurrent candidemia, the *Candida* species or species composition differed between episodes in nine patients (24.3%). A species not detected during the initial episode was recovered during a subsequent episode in five patients (13.5%). In four additional patients (10.8%), one of the two species detected during the initial mixed-species episode was not recovered during the subsequent episode. The same species composition was maintained across all episodes in the remaining 28 patients (75.7%).

For demographic characteristics, clinical variables, mortality outcomes, and logistic regression analyses, each patient was included once using the initial 30-day candidemia episode. For species-specific patient-level analyses, a species was considered involved if it was identified at any time during that initial episode, except that *C. parapsilosis* exposure in the 30-day mortality model was defined by its presence in the index culture. Documented persistent candidemia was assessed across all initial and recurrent episodes, with each *Candida* species evaluated separately from the date of its first isolation within the corresponding episode. Patients meeting the persistence criterion in more than one episode or for more than one *Candida* species were counted only once in the patient-level persistence analysis. The index culture of the initial episode was also used to define the exposure groups for the exploratory comparison of persistence according to the presence or absence of *C. parapsilosis*. Species distribution and antifungal susceptibility analyses were performed at the episode–species level and included both initial and recurrent episodes.

### 3.1. Demographic and Clinical Characteristics

Of the 566 patients, 325 (57.4%) were male and 241 (42.6%) were female. Forty patients (7.1%) were paediatric and 526 (92.9%) were adults. The median age was 67 years (IQR, 53–76). Overall, 418 patients (73.9%) were receiving care in an intensive care unit at the time of the index blood culture.

Cardiovascular disease was the most frequent underlying condition, occurring in 234 patients (41.3%). This was followed by solid malignancy in 174 (30.7%), cerebrovascular disease in 149 (26.3%), diabetes mellitus in 147 (26.0%), respiratory disease in 64 (11.3%), and chronic kidney disease in 51 (9.0%).

Central venous catheterisation, urinary catheterisation, total parenteral nutrition, and mechanical ventilation were present in 417 (73.7%), 495 (87.5%), 221 (39.0%), and 436 patients (77.0%), respectively. Almost all patients had received antibiotics, and 401 patients (70.8%) received antifungal treatment. These demographic and clinical characteristics are summarised in Table 1.

### 3.2. Distribution of Candida Species

Species distribution was evaluated at the episode–species level. For each candidemia episode, the first isolate of each distinct *Candida* species was included. Repeat isolates of the same species within an episode were excluded, whereas different species within the same episode and species recovered during recurrent episodes were counted separately. This approach yielded 637 episode–species isolates from 605 candidemia episodes.

*C. albicans* was the most frequently identified species, accounting for 257 isolates (40.3%). This was followed by *C. parapsilosis* with 218 (34.2%), *C. glabrata* (*Nakaseomyces glabratus*) with 63 (9.9%), and *C. tropicalis* with 63 (9.9%). Overall, non-albicans *Candida* species accounted for 380 of 637 episode–species isolates (59.7%).

Less frequently identified species included *C. krusei* (*Pichia kudriavzevii*) (14/637, 2.2%), *C. kefyr* (*Kluyveromyces marxianus*) (7/637, 1.1%), *C. lusitaniae* (*Clavispora lusitaniae*) (5/637, 0.8%), *C. lipolytica* (*Yarrowia lipolytica*) (3/637, 0.5%), *C. auris* (*Candidozyma auris*) (2/637, 0.3%), *C. famata* (*Debaryomyces hansenii*) (2/637, 0.3%), *C. guilliermondii* (*Meyerozyma guilliermondii*) (2/637, 0.3%), and *C. dubliniensis* (1/637, 0.2%). The complete episode–species distribution is summarised in Table 2.

Selected clinical characteristics according to episode-defined *C. parapsilosis* involvement in the initial candidemia episode are shown in Table 3.

In an exploratory multivariable logistic regression analysis, central venous catheter use (adjusted OR 2.096, 95% CI 1.318–3.332; *p* = 0.002), burn injury (adjusted OR 2.340, 95% CI 1.138–4.813; *p* = 0.021), and ICU care at the time of the index blood culture (adjusted OR 1.648, 95% CI 1.005–2.701; *p* = 0.048) were associated with higher odds of episode-defined *C. parapsilosis* involvement. Total parenteral nutrition was not independently associated (adjusted OR 1.189, 95% CI 0.813–1.739; *p* = 0.373). Given the exploratory and retrospective nature of the analysis, these findings should not be interpreted as causal. The model had a C-statistic of 0.659, and the Hosmer–Lemeshow goodness-of-fit test yielded χ^2^ = 5.919 (df = 8; *p* = 0.656). The full multivariable model, including all nine covariates, is presented in Supplementary Table S1.

### 3.3. Temporal Trends in Candida Species Distribution

The first candidemia episode was recorded on 22 August 2019, meaning that the 2019 data covered only the final months of the year. Data collection continued until May 2024; therefore, the first and final study years represented partial calendar years, and annual episode counts should not be compared directly. In addition, 2019 corresponded to the hospital’s initial operating period, which may also have contributed to the lower number of candidemia episodes recorded during that year.

The annual distribution of the major *Candida* species is presented in Table 4. *C. albicans* and *C. parapsilosis* remained the two leading species throughout the study period. Because the first and final study years were incomplete and multiple episode–species observations could originate from the same patient, temporal differences were described without formal inferential testing. Species distribution did not differ significantly between paediatric and adult patients (*p* = 0.374).

### 3.4. Antifungal Susceptibility Results

Antifungal susceptibility was evaluated for fluconazole, voriconazole, micafungin, and amphotericin B. The analysis was conducted at the episode–species level across 637 episode–species records from 605 candidemia episodes. For each candidemia episode, the susceptibility result of the first isolate of each distinct *Candida* species was included in the analysis. Because susceptibility results were not available for every species–antifungal combination, the number of isolates evaluated differed between antifungal agents. Percentages were calculated using the number of isolates with an available result as the denominator. For *C. parapsilosis*, 218 episode–species isolates were identified, of which 217 had an available fluconazole result; the single isolate without a fluconazole result accounts for the difference between the species total (218) and the fluconazole denominator (217).

Fluconazole resistance was particularly prominent among *C. parapsilosis* isolates. Of the 217 isolates tested, 93 (42.9%) were resistant, 58 (26.7%) were susceptible dose-dependent, and 66 (30.4%) were susceptible. Among the 211 isolates with voriconazole results, seven (3.3%) were resistant and 132 (62.6%) were intermediate. Two of the 217 isolates tested against micafungin (0.9%) were resistant, whereas all 213 isolates with amphotericin B results were classified as WT.

Micafungin and amphotericin B results were available for 63 *C. glabrata* isolates. For micafungin, 61 isolates (96.8%) were susceptible, one (1.6%) was intermediate, and one (1.6%) was resistant. All 63 isolates were WT for amphotericin B. Voriconazole MIC results were available for 45 isolates. Using an epidemiological cutoff value of 0.25 mg/L, 41 isolates (91.1%) were classified as WT and four (8.9%) as non-WT. Voriconazole MIC_50_ and MIC_90_ values were ≤0.12 and 0.25 mg/L, respectively. Fluconazole MIC values for *C. glabrata* were not reported by the VITEK 2 system in the routine laboratory results and were therefore unavailable for retrospective analysis.

Antifungal susceptibility results for other rare *Candida* species were not presented because of the small number of isolates and the absence of CLSI species-specific clinical breakpoints. For *C. krusei*, fluconazole MIC_50_ and MIC_90_ values were not calculated because isolates were reported as intrinsically resistant and individual fluconazole MIC values were not available in the laboratory records. The categorical susceptibility results are summarised in Table 5, and MIC_50_ and MIC_90_ values are presented in Table 6.

Among the 37 patients with recurrent candidemia, at least one *Candida* species was recovered in both the initial and first recurrent episodes in 32 patients. Comparable antifungal susceptibility results for the shared species were available for all 32 patients. No resistant or non-WT phenotype was detected in the shared species in either episode in 24 patients (75.0%). Newly detected resistance in the shared species was observed in the first recurrent episode in three patients (9.4%); in all three, *C. parapsilosis* was recovered in both episodes and the newly detected resistance was to fluconazole. Resistance was detected only in the initial episode in three patients (9.4%), while it was present in both episodes in two patients (6.3%). In one additional patient, species replacement was observed, with fluconazole-susceptible *C. albicans* in the initial episode followed by fluconazole-resistant *C. parapsilosis* in the recurrent episode. This patient was not included in the shared-species susceptibility comparison because the species differed between episodes.

Among the 197 patients with episode-defined *C. parapsilosis* involvement, fluconazole susceptibility results were available for 196. Fifty-eight patients had fluconazole-susceptible isolates, whereas 138 had non-susceptible isolates, including 52 susceptible dose-dependent and 86 resistant isolates. In multivariable logistic regression, age (adjusted OR 1.005, 95% CI 0.992–1.018; *p* = 0.495), central venous catheterisation (adjusted OR 1.166, 95% CI 0.501–2.714; *p* = 0.721), total parenteral nutrition (adjusted OR 0.922, 95% CI 0.479–1.776; *p* = 0.808), and previous gastrointestinal surgery (adjusted OR 0.999, 95% CI 0.400–2.498; *p* = 0.998) were not independently associated with fluconazole non-susceptibility. For this model, the C-statistic was 0.527, and the Hosmer–Lemeshow goodness-of-fit test yielded χ^2^ = 10.994 (df = 8; *p* = 0.202). This C-statistic indicates limited discriminative ability; accordingly, the absence of significant associations in this model should be interpreted with caution. Thirty-day mortality was similar between patients with fluconazole-susceptible and non-susceptible isolates (39.7% vs. 43.5%; OR 1.17, 95% CI 0.63–2.19; *p* = 0.621). Although 60-day mortality was numerically higher in the non-susceptible group (58.0% vs. 48.3%; OR 1.48, 95% CI 0.80–2.74; *p* = 0.213), the difference was not statistically significant.

### 3.5. Mortality Outcomes

All-cause mortality was assessed from the collection date of the index blood culture of the initial candidemia episode. Seven-day mortality occurred in 188 patients (33.2%), 14-day mortality in 252 (44.5%), 30-day mortality in 325 (57.4%), and 60-day mortality in 381 (67.3%).

Neither 30-day nor 60-day mortality differed significantly across the study years (*p* = 0.614 and *p* = 0.824, respectively).

### 3.6. Factors Associated with 30-Day Mortality

In the univariable analyses, increasing age, adult age group, ICU care at the time of the index blood culture, mechanical ventilation, diabetes mellitus, cardiovascular disease, urinary catheterisation, and COVID-19 were associated with higher odds of 30-day mortality. Antifungal treatment, index-defined *C. parapsilosis* presence, burn injury, and trauma were associated with lower odds of 30-day mortality. The associations observed for burn injury and trauma should be interpreted cautiously because they may reflect differences in age and patient population.

In the multivariable analysis, increasing age, ICU care at the time of the index blood culture, mechanical ventilation, and solid malignancy were independently associated with higher odds of 30-day mortality. Each 10-year increase in age was associated with increased odds of mortality (adjusted OR = 1.237; 95% CI: 1.119–1.368; *p* < 0.001). ICU care at the time of the index blood culture (adjusted OR = 2.161; 95% CI: 1.306–3.576; *p* = 0.003) and mechanical ventilation (adjusted OR = 2.366; 95% CI: 1.423–3.934; *p* = 0.001) were also independently associated with increased mortality.

Solid malignancy was independently associated with higher odds of 30-day mortality (adjusted OR = 1.568; 95% CI: 1.019–2.411; *p* = 0.041). Antifungal treatment (adjusted OR = 0.307; 95% CI: 0.195–0.482; *p* < 0.001) and index-defined *C. parapsilosis* presence (adjusted OR = 0.406; 95% CI: 0.272–0.605; *p* < 0.001) were associated with lower odds of 30-day mortality. The model had a C-statistic of 0.755, and the Hosmer–Lemeshow goodness-of-fit test yielded χ^2^ = 7.070 (df = 8; *p* = 0.529). The final multivariable model is shown in Table 7, and cumulative all-cause mortality across follow-up time points is shown in Figure 1.

## 4. Discussion

Although *C. albicans* was the most frequently identified individual species in this study, non-albicans *Candida* species collectively accounted for 59.7% of all isolates. The most notable findings were the high proportion of *C. parapsilosis*, marked fluconazole resistance in this species, recurrent and mixed-species candidemia episodes, and high all-cause mortality.

*C. parapsilosis* accounted for 34.2% of episode–species isolates and ranked second after *C. albicans*. In the ECMM *Candida* III study, its overall proportion was 15%, although it reached approximately 25% in Türkiye and Italy [10]. Studies from Türkiye have also reported *C. parapsilosis* rates ranging from 26.6% to 29% [11,12,13]. The higher proportion observed in our study underscores the variability of candidemia epidemiology across centres and the importance of local surveillance data.

The predominance of intensive care patients and the frequent use of central venous catheters may have contributed to the high proportion of *C. parapsilosis*. This species is well known for its ability to adhere to intravascular surfaces and form biofilms [6,14]. Central venous catheter use has been associated with *C. parapsilosis* candidemia [15,16], while total parenteral nutrition has also been reported as an associated factor [17]. In addition, high glucose concentrations associated with total parenteral nutrition have been shown to enhance biofilm formation [18]. In our exploratory species-specific multivariable analysis, central venous catheter use and burn injury were associated with higher odds of episode-defined *C. parapsilosis* involvement in the initial episode. ICU care at the time of the index blood culture was also associated with higher odds, whereas total parenteral nutrition was not independently associated. These findings may help characterise patients with *C. parapsilosis* candidemia in our centre; however, the exploratory and retrospective nature of the analysis, together with the possibility of residual confounding, precludes causal interpretation.

The most important microbiological finding was the high rate of fluconazole resistance among *C. parapsilosis* isolates. Of the 217 episode–species isolates with available fluconazole results, 42.9% were resistant, 26.7% were susceptible dose-dependent, and only 30.4% were susceptible. Fluconazole MIC_50_ and MIC_90_ values of 4 and 32 µg/mL, respectively, further supported the substantial burden of reduced susceptibility. This resistance rate was markedly higher than the 17% reported in the ECMM Candida III study [10] and the pooled global prevalence of 15.2% reported in a meta-analysis [19]. Because susceptibility was interpreted from VITEK 2 MIC values using CLSI criteria, and clinically important discrepancies between VITEK 2 and the CLSI reference broth microdilution method have been described for certain azole-species combinations [20,21], the resistance rate should be interpreted with this methodological limitation in mind. Nevertheless, the high fluconazole MIC_50_ and MIC_90_ values observed in our cohort were consistent with a substantial burden of reduced susceptibility.

The high burden of fluconazole resistance observed in our cohort also raises the possibility that local clonal dissemination may have contributed to the epidemiological pattern. Clonal outbreaks of fluconazole-resistant *C. parapsilosis*, frequently involving the ERG11 Y132F substitution, have been documented in Türkiye [22,23]. However, the associations with central venous catheter use and burn injury in our study were observed for *C. parapsilosis* involvement rather than for fluconazole resistance itself, and molecular typing and resistance-mechanism analyses were not performed. Therefore, our data cannot distinguish clonal transmission from antifungal selection or other hospital-level factors. Genotyping of contemporary *C. parapsilosis* bloodstream isolates would help clarify this possibility. Mechanistic approaches that directly interrogate antifungal targets may complement phenotypic susceptibility testing and help clarify the molecular basis of resistance in future studies [24].

A multicentre study from Türkiye reported fluconazole resistance in 26.7% of *C. parapsilosis* isolates and also identified echinocandin-resistant and multidrug-resistant isolates [22]. In a study conducted in Bursa, fluconazole non-susceptibility was associated with older age, central venous catheter use, total parenteral nutrition, and colostomy [25]. In our study, age, central venous catheter use, total parenteral nutrition, and previous gastrointestinal surgery were not significantly associated with fluconazole non-susceptibility. However, colostomy represents a more specific exposure than the broader variable of previous gastrointestinal surgery used in our study, and these variables should not be considered directly equivalent.

Among 196 patients with episode-defined *C. parapsilosis* involvement in the initial episode and an available fluconazole result, 30-day mortality was similar between the fluconazole-susceptible and non-susceptible groups (OR 1.17, 95% CI 0.63–2.19). Although 60-day mortality was numerically higher in the non-susceptible group, the estimate remained imprecise and was not statistically significant (OR 1.48, 95% CI 0.80–2.74). Similarly, a recent multicentre study found no independent association between fluconazole resistance and mortality among appropriately treated patients, although resistance was associated with a higher risk of recurrence [26]. Therefore, the clinical significance of fluconazole resistance is not limited to mortality, as it may restrict empirical treatment options; previous studies have also suggested an association with recurrent infection.

Antifungal resistance was less common among the other species. Azole resistance was rare among *C. albicans* isolates, and micafungin susceptibility was largely preserved. Although *C. parapsilosis* is known to exhibit intrinsically higher echinocandin MICs than many other *Candida* species [14], echinocandin resistance has generally remained uncommon in published studies [6]. A similar pattern was observed in our cohort: micafungin MIC_50_ and MIC_90_ values for *C. parapsilosis* were 0.5 and 1 µg/mL, respectively, higher than those observed for *C. albicans*, *C. tropicalis*, and *C. glabrata*, whereas only 2 of 217 *C. parapsilosis* isolates (0.9%) were resistant to micafungin. Nevertheless, the emergence of echinocandin-resistant isolates reported from Türkiye [22] supports continued species-level identification and antifungal susceptibility testing.

Because no CLSI clinical breakpoint is available for voriconazole against *C. glabrata*, the results were interpreted using WT/non-WT terminology. Although studies have generally reported good agreement between VITEK 2 and the CLSI reference broth microdilution method, clinically important discrepancies may occur, particularly with certain azole-species combinations [20,21]. Unexpected resistance or non-WT results should therefore be confirmed using a reference method whenever possible.

The IDSA guideline and the current ECMM-ISHAM-ASM global guideline recommend an echinocandin as initial treatment for most adults with candidemia [5,27]. Given the high frequency of fluconazole-resistant *C. parapsilosis* in our centre, empirical fluconazole therapy before species identification and susceptibility results become available may not be appropriate, particularly in critically ill patients. Transition to azole therapy should be guided by documented susceptibility results. Although current guidelines favour echinocandins as initial therapy, other approaches are also being investigated for difficult-to-treat fungal infections. These include the repurposing of existing drugs and nanotechnology-based treatment strategies, which may eventually expand the therapeutic options for resistant *Candida* infections, although their clinical role remains to be established [28].

Recurrent candidemia was identified in 37 patients, accounting for 39 recurrent episodes. The species or species composition differed between episodes in nine patients, while a species not detected during the initial episode was isolated in a subsequent episode in five patients. In the susceptibility comparison of shared species, newly detected fluconazole resistance was observed in the first recurrent episode in three patients. In all three patients, *C. parapsilosis* was isolated in both the initial and recurrent episodes. In one additional patient, fluconazole-susceptible *C. albicans* was isolated in the initial episode, whereas fluconazole-resistant *C. parapsilosis* was detected in the recurrent episode. Because the species differed between episodes, this patient was not included in the shared-species susceptibility comparison. As molecular typing was not performed, newly detected resistance in patients with the same species in both episodes cannot be conclusively interpreted as resistance acquired over time by the same strain. A population-based study from the United States reported recurrent candidemia in approximately 6% of patients, with a different *Candida* species identified in 42% of patients when the first and second episodes were compared [29]. These findings suggest that recurrent candidemia may not always represent relapse of the initial infection and support repeating species identification and antifungal susceptibility testing in each new episode.

Using a culture-date-based definition applied separately to each *Candida* species within all initial and recurrent candidemia episodes, documented persistent candidemia was observed in 128 patients (22.6%). At the patient–species level, *C. parapsilosis* and *C. albicans* were the most frequently involved persistent species, occurring in 54 and 53 patients, respectively. Five patients met the persistence criterion for two different *Candida* species. Persistence was significantly more frequent among patients whose index culture of the initial episode included *C. parapsilosis* than among those whose index culture did not. However, the association was modest and should be interpreted cautiously because the confidence interval was close to the null value and the analysis was exploratory. The overall persistence proportion was close to the 25.1% reported in the recent multicentre study by Tiseo et al. [30]; however, direct comparison is limited because that study defined persistence from the start of active antifungal therapy and included only patients with available follow-up blood cultures. In our study, exact treatment initiation dates and systematically obtained negative follow-up blood cultures were unavailable; therefore, our estimate should be interpreted as documented microbiological persistence based on culture dates rather than persistence defined in relation to active antifungal therapy. Recent multicentre data identified *C. parapsilosis*, total parenteral nutrition, and *C. auris* as factors associated with persistent candidemia, whereas early source control was associated with a lower risk of persistence [30]. Together with current recommendations for follow-up blood cultures [27], these findings underscore the importance of repeated blood cultures and timely assessment for source control.

Different *Candida* species were identified from the same blood culture barcode or within the same episode in 32 patients. Similar mixed-species episodes have been reported in previous surveillance studies [31]. Patient-level analyses prevented the same patient from being counted more than once in clinical and mortality analyses, whereas the episode–species approach preserved the microbiological characteristics of recurrent and mixed-species episodes.

Two *C. auris* episodes were identified in our study. Although the number was small, detection of this species was epidemiologically important because *C. auris* is classified as a critical-priority fungal pathogen owing to its environmental persistence, identification difficulties, and antifungal resistance [32].

All-cause mortality was 57.4% at 30 days and 67.3% at 60 days. In the multivariable model, older age, ICU care at the time of the index blood culture, mechanical ventilation, and solid malignancy were associated with higher odds of 30-day mortality. Thirty-day all-cause mortality in our cohort (57.4%) was higher than the 33.9% 30-day mortality reported among candidemia cases in the ECMM *Candida* III matched cohort [33] and the 42% crude 30-day mortality reported in the EUCANDICU study [34]. In contrast, the 56.7% 30-day mortality reported in a multicentre Turkish study was very similar to that observed in our cohort [35]. However, the retrospective data did not allow deaths attributable to candidemia to be distinguished from those related to underlying diseases or ongoing critical illness.

Although antifungal treatment and the presence of *C. parapsilosis* in the index culture were associated with lower odds of 30-day mortality in the multivariable model, these findings should not be interpreted as causal or intrinsically protective. Treatment timing, disease severity, differences between patient populations, and residual confounding could not be fully addressed. The association between antifungal treatment and lower mortality may also have been influenced by immortal-time bias, because treatment was analysed as a binary exposure and exact treatment initiation times were unavailable. Unmeasured differences in infection source and source-control practices, including catheter removal, may also have contributed to this association.

The strengths of this study include the large number of patients and the separate evaluation of patient-level clinical findings and episode–species level microbiological data. The main limitations were the retrospective, single-centre design, incomplete clinical and susceptibility data, lack of detailed information on the source of infection and timing of treatment, absence of reference susceptibility testing, and lack of molecular typing. Species identification relied on routine phenotypic and automated methods without molecular confirmation. Therefore, species misidentification cannot be excluded, particularly among uncommon *Candida* species, for which limitations of conventional and automated identification methods have been reported [36]. Exact antifungal treatment initiation dates and systematic negative follow-up blood cultures were unavailable. Accordingly, persistent candidemia was assessed using a culture-date-based definition applied separately to each *Candida* species across all candidemia episodes. Because follow-up blood cultures were not obtained according to a standardised schedule, documented persistence depended on culture sampling and may have been underestimated; bloodstream clearance could not be evaluated. The species-specific models were exploratory, and their findings should be interpreted in light of possible residual confounding and multiple comparisons.

A previously published study from our centre evaluated a partially overlapping subgroup restricted to catheter-related candidemia between January 2021 and December 2025 [37]. That study used a treatment-based definition of persistence (blood cultures remaining positive for at least 72 h despite antifungal therapy) and focused on catheter management, host inflammatory markers, recatheterisation, and secondary bacterial bloodstream infections. In contrast, the present hospital-wide cohort includes candidemia episodes identified between August 2019 and May 2024 and uses a culture-date-based definition of documented microbiological persistence, while focusing on epidemiology, temporal and species distribution, antifungal susceptibility, recurrent and mixed-species episodes, and mortality. No tables, figures, or statistical models from the previous publication were reproduced in the present study.

## 5. Conclusions

In this large single-centre cohort, non-albicans *Candida* species predominated, driven by a high burden of *C. parapsilosis*, nearly half of which were resistant to fluconazole. Documented microbiological persistence was identified in 22.6% of the overall cohort. Among patients with recurrent candidemia, species composition and antifungal susceptibility profiles could differ between episodes. Together with a 30-day mortality exceeding 50%, these findings show that candidemia in our centre is characterised by a combination of substantial mortality, persistence, recurrence, and clinically important antifungal resistance. Empirical reliance on fluconazole may therefore be inappropriate in settings with similar epidemiology; treatment decisions should be informed by local surveillance, species-level identification, repeat blood cultures, and episode-specific antifungal susceptibility testing.

## Figures and Tables

**Figure 1:**
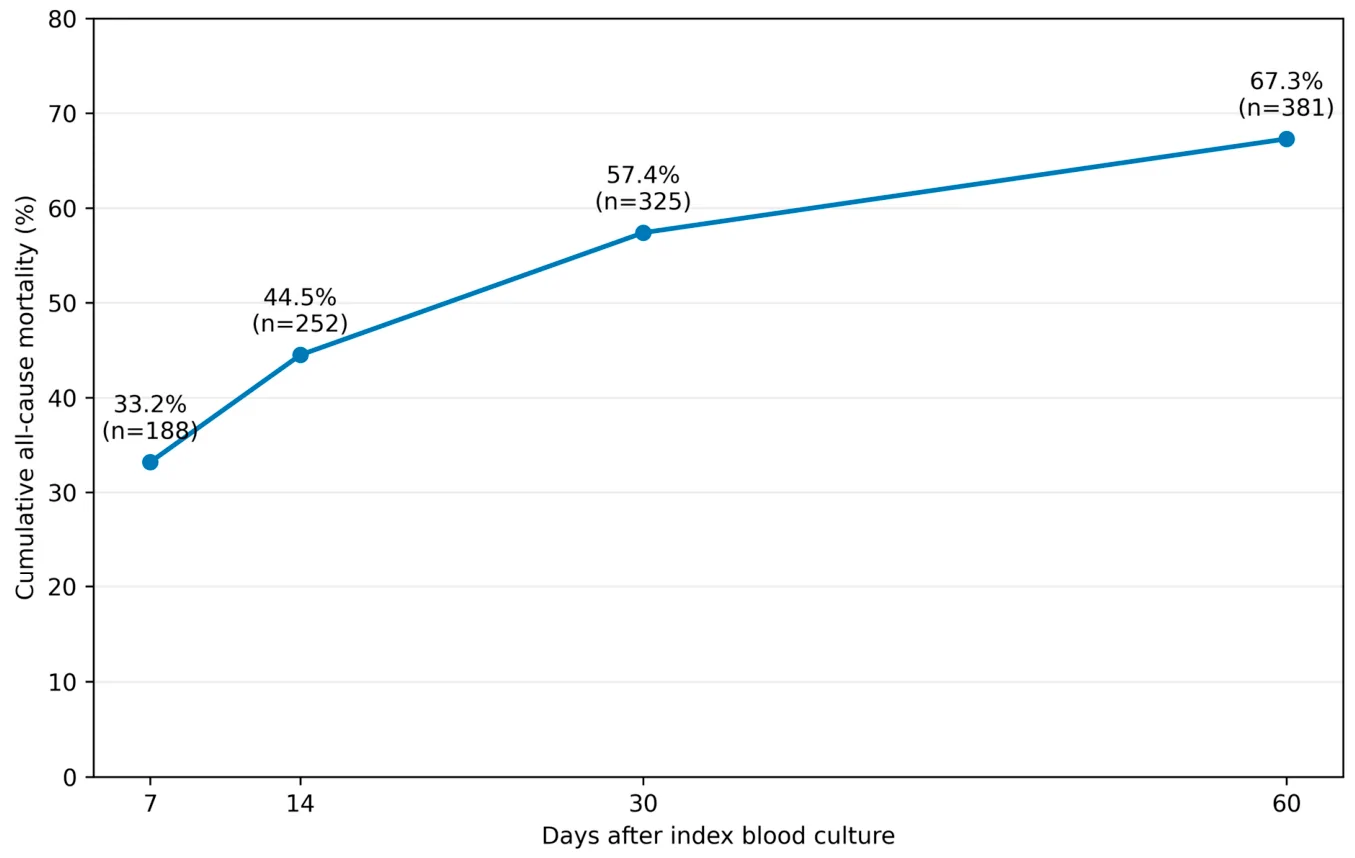
Cumulative all-cause mortality at 7, 14, 30, and 60 days after the index blood culture.

**Table 1:** Demographic and clinical characteristics of patients with candidemia.

Characteristic	Patients, *n* = 566
Age, years, median (IQR)	67 (53–76)
Male sex	325 (57.4)
Adult patients	526 (92.9)
ICU care at index blood culture	418 (73.9)
**Underlying diseases/comorbidities**	
Cardiovascular disease	234 (41.3)
Solid malignancy	174 (30.7)
Cerebrovascular disease	149 (26.3)
Diabetes mellitus	147 (26.0)
Respiratory disease	64 (11.3)
Chronic liver disease	5 (0.9)
Chronic kidney disease	51 (9.0)
Haematological malignancy	25 (4.4)
Prematurity	8 (1.4)
Other comorbidities	22 (3.9)
**Clinical conditions and invasive procedures**	
COVID-19	70 (12.4)
Burn injury	41 (7.2)
Trauma	20 (3.5)
Previous gastrointestinal surgery	93 (16.4)
PEG	50 (8.8)
Central venous catheter	417 (73.7)
Urinary catheter	495 (87.5)
Total parenteral nutrition	221 (39.0)
Mechanical ventilation	436 (77.0)
Antibiotic exposure	564 (99.6)
Antifungal treatment	401 (70.8)

Data are presented as *n* (%) unless otherwise indicated. ICU, intensive care unit; IQR, interquartile range; PEG, percutaneous endoscopic gastrostomy. Bold text indicates category headings.

**Table 2:** Distribution of *Candida* species across all candidemia episodes at the episode–species level.

Species	*n* (%)	Species	*n* (%)	Species	*n* (%)
*C. albicans*	257 (40.3)	*C. glabrata*	63 (9.9)	*C. lipolytica*	3 (0.5)
*C. parapsilosis*	218 (34.2)	*C. tropicalis*	63 (9.9)	*C. auris*	2 (0.3)
*C. krusei*	14 (2.2)	*C. kefyr*	7 (1.1)	*C. famata*	2 (0.3)
*C. lusitaniae*	5 (0.8)	*C. guilliermondii*	2 (0.3)	*C. dubliniensis*	1 (0.2)

Data are presented as *n* (%); total *n* = 637.

**Table 3:** Comparison of selected clinical characteristics according to episode-defined *C. parapsilosis* involvement in the initial candidemia episode.

Variable	Episode-Defined *C. parapsilosis* Involvement, *n* = 197	No Episode-Defined *C. parapsilosis* Involvement, *n* = 369	*p* Value
Central venous catheter use	166/197 (84.3%)	251/369 (68.0%)	<0.001
Total parenteral nutrition	81/197 (41.1%)	140/369 (37.9%)	0.461
ICU care at index blood culture	157/197 (79.7%)	261/369 (70.7%)	0.021
Burn injury	23/197 (11.7%)	18/369 (4.9%)	0.003

Episode-defined *C. parapsilosis* involvement was defined as identification of *C. parapsilosis* at any time during the initial 30-day candidemia episode. Data are presented as n/N (%). *p* values were calculated using the chi-square test or Fisher’s exact test, as appropriate. ICU, intensive care unit. The denominators in Table 3 (197 vs. 369) are based on episode-defined *C. parapsilosis* involvement, whereas the persistence comparison in Section 3 (192 vs. 374) was based on *C. parapsilosis* presence in the index blood culture.

**Table 4:** Distribution of major *Candida* species by study period at the episode–species level.

Study Period	Total *n*	*C. albicans* *n* (%)	*C. parapsilosis* *n* (%)	*C. glabrata* *n* (%)	*C. tropicalis* *n* (%)	Other *n* (%)
2019 Aug–Dec	19	12 (63.2)	6 (31.6)	0 (0.0)	1 (5.3)	0 (0.0)
2020	85	28 (32.9)	35 (41.2)	6 (7.1)	10 (11.8)	6 (7.1)
2021	124	43 (34.7)	45 (36.3)	7 (5.6)	21 (16.9)	8 (6.5)
2022	150	57 (38.0)	57 (38.0)	17 (11.3)	12 (8.0)	7 (4.7)
2023	184	90 (48.9)	50 (27.2)	22 (12.0)	9 (4.9)	13 (7.1)
2024 Jan–May	75	27 (36.0)	25 (33.3)	11 (14.7)	10 (13.3)	2 (2.7)
Total	637	257 (40.3)	218 (34.2)	63 (9.9)	63 (9.9)	36 (5.7)

Data are presented as *n* (%), with percentages calculated within each study period. Data collection covered August–December in 2019, the full calendar years from 2020 to 2023, and January–May in 2024. Therefore, absolute counts for 2019 and 2024 should not be directly compared with those of the complete calendar years. “Other” includes all *Candida* species other than *C. albicans*, *C. parapsilosis*, *C. glabrata*, and *C. tropicalis*.

**Table 5:** Antifungal susceptibility results at the episode–species level.

Species	Agent	Tested, *n*	S, *n* (%)	WT, *n* (%)	I, *n* (%)	SDD, *n* (%)	R, *n* (%)	Non-WT, *n* (%)
*C. albicans*	FLC	257	255 (99.2)	–	–	0	2 (0.8)	–
	VRC	256	255 (99.6)	–	0	–	1 (0.4)	–
	MCF	257	257 (100)	–	0	–	0	–
	AMB	256	–	256 (100) ^a^	–	–	–	0
*C. parapsilosis*	FLC	217	66 (30.4)	–	–	58 (26.7)	93 (42.9)	–
	VRC	211	72 (34.1)	–	132 (62.6)	–	7 (3.3)	–
	MCF	217	215 (99.1)	–	0	–	2 (0.9)	–
	AMB	213	–	213 (100) ^a^	–	–	–	0
*C. tropicalis*	FLC	63	60 (95.2)	–	–	3 (4.8)	0	–
	VRC	63	63 (100)	–	0	–	0	–
	MCF	63	63 (100)	–	0	–	0	–
	AMB	63	–	63 (100) ^a^	–	–	–	0
*C. glabrata*	VRC	45	–	41 (91.1) ^a^	–	–	–	4 (8.9) ^a^
	MCF	63	61 (96.8)	–	1 (1.6)	–	1 (1.6)	–
	AMB	63	–	63 (100) ^a^	–	–	–	0
*C. krusei*	VRC	14	14 (100)	–	0	–	0	–
	MCF	14	12 (85.7)	–	2 (14.3)	–	0	–
	AMB	12	–	12 (100) ^a^	–	–	–	0

Abbreviations: FLC, fluconazole; VRC, voriconazole; MCF, micafungin; AMB, amphotericin B; S, susceptible; SDD, susceptible dose-dependent; I, intermediate; R, resistant; WT, wild type; non-WT, non-wild type. Data are presented as *n* (%) unless otherwise indicated. Tested, n indicates the number of episode–species isolates with an available susceptibility result for the corresponding antifungal agent. Differences in denominators between agents reflect unavailable susceptibility results in the retrospective laboratory records. A dash (–) indicates that the corresponding interpretive category is not applicable to that species–antifungal combination, whereas 0 indicates an applicable category in which no isolate was classified. ^a^ Voriconazole results for *C. glabrata* and amphotericin B results were interpreted as WT/non-WT based on CLSI epidemiological cutoff values. Fluconazole results are not shown for *C. krusei* because of its intrinsic resistance to fluconazole.

**Table 6:** MIC_50_ and MIC_90_ values by antifungal agent.

Species	Antifungal Agent	Tested, *n*	MIC_50_	MIC_90_
*C. albicans*	Fluconazole	257	≤0.5	≤0.5
	Voriconazole	256	≤0.12	≤0.12
	Micafungin	257	≤0.06	≤0.06
	Amphotericin B	256	0.5	1
*C. parapsilosis*	Fluconazole	217	4	32
	Voriconazole	211	0.25	0.5
	Micafungin	217	0.5	1
	Amphotericin B	213	0.5	1
*C. tropicalis*	Fluconazole	63	1	2
	Voriconazole	63	≤0.12	≤0.12
	Micafungin	63	≤0.06	≤0.06
	Amphotericin B	63	≤0.25	0.5
*C. glabrata*	Voriconazole	45	≤0.12	0.25
	Micafungin	63	≤0.06	≤0.06
	Amphotericin B	63	0.5	1
*C. krusei*	Voriconazole	14	≤0.12	0.25
	Micafungin	14	0.12	0.5
	Amphotericin B	12	0.5	1

Abbreviations: MIC, minimum inhibitory concentration; MIC_50_ and MIC_90_, minimum inhibitory concentrations inhibiting 50% and 90% of isolates, respectively. Tested, n indicates the number of episode–species isolates with an available susceptibility result for the corresponding antifungal agent. Differences in denominators between agents reflect unavailable susceptibility results in the retrospective laboratory records. MIC values are expressed in mg/L.

**Table 7:** Multivariable logistic regression analysis of factors associated with 30-day mortality.

Variable	Adjusted OR	95% CI	*p* Value
Age, per 10-year increase	1.237	1.119–1.368	<0.001
ICU care at index blood culture	2.161	1.306–3.576	0.003
Mechanical ventilation	2.366	1.423–3.934	0.001
Solid malignancy	1.568	1.019–2.411	0.041
Antifungal treatment	0.307	0.195–0.482	<0.001
Index-defined *C. parapsilosis* presence	0.406	0.272–0.605	<0.001

CI, confidence interval; ICU, intensive care unit; OR, odds ratio.

## Data Availability

The data supporting the findings of this study are not publicly available because they contain patient-level information and are subject to confidentiality restrictions. An anonymised dataset may be made available from the corresponding author on reasonable request and after approval by the relevant institution and ethics committee.

## Supplementary Materials

The following supporting information can be downloaded at: https://www.mdpi.com/article/10.3390/jof12100715/s1. Table S1. Multivariable logistic regression analysis of factors associated with episode-defined *Candida* *parapsilosis* involvement in the initial candidemia episode.

## Abbreviations

ASM	American Society for Microbiology
ATCC	American Type Culture Collection
CI	confidence interval
CLSI	Clinical and Laboratory Standards Institute
COVID-19	coronavirus disease 2019
CVC	central venous catheter
ECMM	European Confederation of Medical Mycology
ECV	epidemiological cutoff value
EMB	eosin methylene blue
ICU	intensive care unit
IDSA	Infectious Diseases Society of America
IQR	interquartile range
ISHAM	International Society for Human and Animal Mycology
MIC	minimum inhibitory concentration
MIC_50_	minimum inhibitory concentration inhibiting 50% of isolates
MIC_90_	minimum inhibitory concentration inhibiting 90% of isolates
OR	odds ratio
PEG	percutaneous endoscopic gastrostomy
SDD	susceptible dose-dependent
TPN	total parenteral nutrition
WT	wild type
non-WT	non-wild type
