# Supplementary material for: Epidemiological Characteristics, Species Distribution, and Antifungal Resistance Profiles of Candidemia Cases
Source: J Fungi (Basel). 2026 Sep 23;12(10):715. doi: 10.3390/jof12100715 (PMC13706330; doi:10.3390/jof12100715)
Supplement: Supplementary file 1 [file jof-12-00715-s001.zip › jof-4539050-supplementary.pdf]

Supplementary Materials

Manuscript ID: jof-4539050 **Title:** Epidemiological Characteristics, Species Distribution, and Antifungal Resistance Profiles of Candidemia Cases

Table S1. Multivariable logistic regression analysis of factors associated with episode-defined *Candida parapsilosis* involvement in the initial candidemia episode.

**Variable**	**Adjusted OR**	**95% CI**	**p value**
Age, per year	0.994	0.985–1.004	0.244
Male sex	1.361	0.940–1.971	0.103
ICU care at index blood culture	1.648	1.005–2.701	0.048
Total parenteral nutrition	1.189	0.813–1.739	0.373
PEG	1.571	0.841–2.935	0.156
Burn injury	2.340	1.138–4.813	0.021
Solid malignancy	0.878	0.566–1.364	0.563
Cerebrovascular disease	1.271	0.825–1.958	0.278
Central venous catheter use	2.096	1.318–3.332	0.002

Abbreviations: OR, odds ratio; CI, confidence interval; ICU, intensive care unit; PEG, percutaneous endoscopic gastrostomy.

Episode-defined *C. parapsilosis* involvement was defined as identification of *C. parapsilosis* at any time during the initial 30-day candidemia episode (n = 197 with involvement; n = 369 without; total n = 566). Age was entered as a continuous variable. Female sex and absence of each binary clinical characteristic were used as reference categories. Model discrimination: C-statistic 0.659. Calibration: Hosmer-Lemeshow goodness-of-fit test $\chi^2 = 5.919$ (df = 8; p = 0.656).
